# Protocols to co-culture human primary lung cells in the simple-flow device

**DOI:** 10.1016/j.xpro.2025.103892

**Published:** 2025-06-17

**Authors:** Cinta Iriondo, Sem Koornneef, Kari-Pekka Skarp, Marjon Buscop-van Kempen, Anne Boerema-de Munck, Robbert J. Rottier

**Affiliations:** 1 Department of Pediatric Surgery, Sophia Children’s Hospital, Erasmus Medical Center, Rotterdam, the Netherlands; 2 Department of Cell Biology, Erasmus Medical Center, Rotterdam, the Netherlands

**Keywords:** Cell culture, Cell isolation, Biotechnology and bioengineering

## Abstract

Human lung models replicate various aspects to address diverse research questions. The complexity of human lung models, such as co-cultures and lung-on-chip devices, is increasing, but details on culture methodologies are often lacking. Here, we describe steps for the isolation, maintenance, and co-culturing of primary epithelial, endothelial, and mesenchymal cells derived from human lung resection material. We then detail procedures for 3D printing the simple-flow device, setting it up with co-cultures of human primary epithelial and endothelial cells under fluidic conditions.

## Highlights

• Protocol to isolate and maintain human primary lung cells
• Instructions for establishing 2D and 3D tri-cultures with human primary lung cells
• Steps to manufacture and generate lung cultures using chip-like simple-flow device

## Abstract (editor highlights)

Publisher’s note: Undertaking any experimental protocol requires adherence to local institutional guidelines for laboratory safety and ethics.

## Graphical abstract

**Figure:**
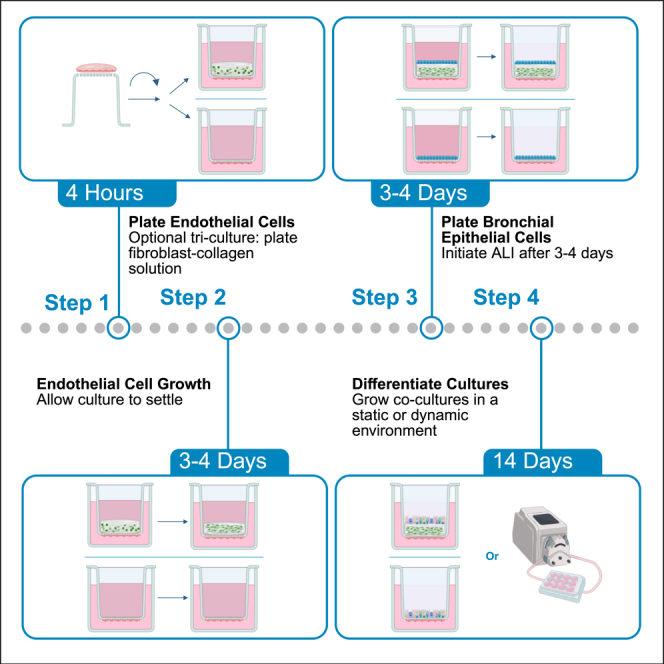

## Before you begin

Current animal and *in vitro* models inadequately recapitulate the human lung characteristics, leading to suboptimal predictive outcomes for potential respiratory treatments and patient-specific situations. To address this limitation, extensive efforts are directed towards refining *in vitro* models that more accurately mimic the human lung physiology, such as differentiated epithelial cultures assessed for barrier function and ciliary activity,^1^^,^^2^^,^^3^^,^^4^^,^^5^ and precision-cut lung slices (PCLS),^6^^,^^7^ organ-on-chip platforms and 3D airway cultures.^8^^,^^9^^,^^10^^,^^11^ Within this evolving landscape, our co- and tri-culture systems offer a platform to also study lung biology. Here, we provide a comprehensive protocol for establishing co-cultures of human primary bronchial epithelial cells (hPBECs) and human lung microvascular endothelial cells (hMVECs), and of three-dimensional tri-cultures comprising human lung fibroblasts (hLFs) embedded in collagen matrices, alongside hPBECs and hMVECs. In the described configuration, the hPBECs are air-exposed to develop a differentiated muco-ciliary epithelium, hLFs are embedded in a type-I collagen matrix mimicking the lung submucosa, and hMVECs are plated on the basolateral side of membranes to be exposed to flow medium. Finally, we elaborate on manufacturing, post-processing, and setting up steps for a 3D-printed, biocompatible flow cell culture plated – termed Simple-Flow – that allows the integration of commercial cell culture inserts containing the lung co-cultures, and medium flow in the basal chamber.^12^ Protocols for isolating and culturing of primary hPBECs, hMVECs and hLFs from human lung resection material are described at the end of the manuscript.

### Prepare the workspace

**Timing: 0–7 days**

Before putting co-cultures or tri-cultures at air-liquid interface (ALI), ensure that a couple Simple-Flow devices are already 3D-printed, post-processed and ready to set up. In a cell culture cabinet and under sterile conditions.

1. Thaw the required cell types needed for setting up co- or tri-cultures.

***Note:*** The different cell types do not grow equally well, so plan accordingly the timing of initiating the individual cultures (see Table below for details).

**Table:** 

Cell type	Grown until confluency	Number of cells frozen per vial	Number of days to reach desired confluency for 10 cm dishes
hPBECs	95–98%	0.4–0.5 × 10^6^ cells	6-7 days
hMVECs	95%	0.35–0.4 × 10^6^ cells	6-7 days
hLFs	30–50%	0.3 × 10^6^ cells	5 days

2. On the day to set up the co- or tri-cultures, the cells need to be sufficiently confluent to have enough cells.

***Note:*** For standard cell maintenance, we usually grow primary cells up to about 85% confluency to avoid contact inhibition. However, when we grow primary cells before setting up ALI cultures, we usually leave hMVECs and hPBECs to grow up to 95% confluent to maximize cell yield as we require many cells, and hLFs to 30-50% confluency as lower numbers of hLFs are needed. It is crucial to prevent hPBECs from overgrowing (over 98% confluent), as this can lead high cell death rates during trypsinization. Therefore, once the cells are fully confluent, cells should be harvested within the next 24 h. Co-cultures and tri-cultures are preferably established using cells derived from the same donor. When this is not feasible, cells from donors of the same sex are paired to minimize biological variability. Cells from different donors were not pooled together.

3. Pre-warm cell culture media and trypsin to 37°C in a water bath 30 min.
4. Make sure to have a bucket of ice for items that need to be kept cold, such as coatings.
5. Clean surfaces of a cell culture cabinet with 70% ethanol (EtOH).
6. Ensure to have all items needed beforehand, sterilize them (also with 70% EtOH), and place them into the cell culture cabinet.
7. After all preparations are completed, transfer the cells into the cell culture cabinet and start the procedures.

### Institutional permissions

Lung tissue was obtained from residual, tumor-free material obtained at lung resection surgery for lung cancer. The Medical Ethical Committee of the Erasmus MC Rotterdam granted permission for this study (METC 2012–512).

## Key resources table

**Table:** 

REAGENT or RESOURCE	SOURCE	IDENTIFIER
**Antibodies**		

CD31-Dynabeads	Thermo Fisher Scientific, Invitrogen	11155D
CD31, mouse (1:100)	BioLegend	303101
ERG1, rabbit (1:500)	Abcam	ab92513
MUC5AC, mouse (1:500)	Abcam	ab3649
MUC5B, rabbit (1:500)	Sigma-Aldrich	HPA008246
P63, mouse (1:50)	Abcam	ab735
TUBIV, mouse (1:200)	Biogenex	MU178-UC
VIM, rabbit (1:500)	Abcam	ab92547
ZO1, rabbit (1:100)	Zymed	40–2300
Alexa Fluor 488 Donkey anti-mouse IgG (1:500)	Jackson ImmunoResearch	715-545-151
Alexa Fluor 594 Donkey anti-rabbit IgG (1:500)	Jackson ImmunoResearch	711-585-152

**Biological samples**		

Healthy human lung tissue	Pathology Department, Erasmus Medical Center (EMC), Rotterdam, NL	N/A
Healthy human bronchus ring	Pathology Department, EMC, Rotterdam, NL	N/A

**Chemicals, peptides, and recombinant proteins**		

Protease type XIV, ≥ 3.5 units/mg solid, powder	Sigma-Aldrich	P5147-1G
Isoprotenerol (IP)	Sigma-Aldrich	I-6504-100 mg
Epidermal growth factor (EGF)	Thermo Fisher Scientific	10450–013
Bovine pituitary extract (BPE) for KSFM medium	Thermo Fisher Scientific	13028–014
BPE + EGF (Kit)	Thermo Fisher Scientific	37000–015
KSFM with L-glutamine	Thermo Fisher Scientific	1 × 500 mL: 17005-034 10 × 500 mL: 17005-059
KSFM with L-glutamine + BPE + EGF (Kit)	Thermo Fisher Scientific	17005–042
Difco Trypsin 1:250	BD Biosciences	215240– 100g
EDTA	Sigma-Aldrich	E1644-1KG
D-(+)-glucose	Sigma-Aldrich	G6152-100G
Soy Bean Trypsin Inhibitor (SBTI)	Sigma-Aldrich	T9128-1G
EC-23 (retinoic acid agonist)	Tocris	4011, 10 mg
BEpiCM medium, 500 mL	ScienCell	3211
DMEM medium, 500 mL with 4.5 g glucose	STEMCELL Technologies	36250
BEpiCGS (supplement)	STEMCELL Technologies	3262
1 M HEPES buffer solution	Gibco, Thermo Fisher Scientific	15630–056
Collagenase type I	Worthington Biochemical	LS004196, 1g
Dispase I, neutral protease, grade I	Roche	04942086001
Collagen R solution (0.4%)	Serva	47256.01
Glacial acetic acid in sterile water	Fisher Scientific	505216, 17.4 N
EGM-2MV medium	Lonza	CC-3202
DMEM (1×), high glucose, pyruvate	Gibco, Thermo Fisher Scientific	41966–029
Fetal bovine serum (FBS)	Capricorn Scientific	FBS-12A
100× Penicillin and Streptomycin (P/S)	Sigma-Aldrich	P0781
DPBS (1×)	Sigma-Aldrich	D8537-500ML
PureCol, 3 mg/mL	Advanced Biomatrix	5005
Bovine serum albumin (BSA)	Sigma-Aldrich	A7030-10g
Human fibronectin, 5 mg, stock 1 mg/mL	EDM Millipore	FC010
Trypsin-EDTA (TE) solution	Sigma-Aldrich	T3924
Dimethyl sulfoxide (DMSO)	Merck, Sigma-Aldrich	1029500500
DPBS + CaCl_2_ + MgCl_2_	Gibco, Thermo Fisher Scientific	14287-072, 1L
ddH_2_O (sterile)	EMC	N/A
GA-1000	Lonza	CC-4083
Cellmatrix Type I -A (type I collagen solution)	Nitta Gelatin	631–00651
Reconstitution buffer	Nitta Gelatin	635–00791
MEM (10×), no glutamine	Gibco, Thermo Fisher Scientific	11430–030
2-Propanol (Isopropanol), ≥ 99.8%	Sigma-Aldrich	59300-2.5L

**Other**		

Magnetic Particle Concentrator (Dynal MPC-S)	Dynal Biotech Thermo Fisher Scientific	120.20 A13346
Falcon 5 mL flow cytometry tubes with cell strainer snap cap	Falcon, Corning	352235
Sterile 100 μm cell strainer	Falcon, Corning	352360
15 mL Cellstar tubes	Greiner Bio-One	188271-N
50 mL Cellstar tubes	Greiner Bio-One	227270
0.2 μm Puradisc FP 30 mm cellulose acetate syringe filter	Whatman, GE Healthcare	10462200
6-well Cellstar cell culture plate	Greiner Bio-One	657160
100 mm tissue culture dish	Sarstedt	83.3902
60 mm tissue culture dish	Falcon, Corning	353004
12 mm Transwell with 0.4 μm pore PET membrane insert	Costar, Corning	3460
6.5 mm Transwell with 0.4 μm pore PET membrane insert	Costar, Corning	3470
6.5 mm CELLTREAT 0.4 μm polyethylene membrane inserts	CELLTREAT, STEMCELL Technologies	100–0997
EVOM2 epithelial voltohmmeter (TEER machine)	World Precision Instruments (WPI)	N/A
STX2 electrode (for TEER machine)	WPI	N/A
Tweezers, Inox	Fine Science Tools (FST) by Dumont, Switzerland	11252–20
Carbon steel surgical blade	Swann-Morton	0103
Disposable scalpel (sterile)	Swann-Morton	0501
Scissors, stainless steel	Medicon, Germany	2528 02.06.10
BioMed Clear Resin Cartridge 1 L	Formlabs	RS-F2-BMCL-01
Form 3B+ 3D Printer	Formlabs	F3B-P-PRINTER
Form 3 Resin Tank v.2.1	Formlabs	RT-F3-02-01
Build Platform (Form 3)	Formlabs	BP-F3-01
FormWash	Formlabs	FH-WA-01
FormCure	Formlabs	FH-CU-01
Surgical disposable scalpel	AESCULAP	5518059
Three-stop Pharmed BPT tubing, 1.02 mm ID	Masterflex, Ismatec, Fisher Scientific Ismatec, Imlab	95714–28 SC0309
Pharmed BPT extension tubing, 1.02 mm ID, 3 meters	Masterflex, Ismatec, Fisher Scientific Ismatec, Imlab	95809–28 SC0343
Luer lock connector male	ibidi	10826
Luer lock connector female	ibidi	10825
Peristaltic pump for incubator G100-1J (6 rollers, 8 channels)	Longer, Darwin Microfluidics	LG-G100-1J-EU/DG-8-A
DOW Corning high vacuum grease	Sigma-Aldrich	Z273554-1EA

## Materials and equipment

**Table:** Bovine serum albumin (BSA)

Reagent	Final concentration	Amount
BSA powder	1 mg/mL (stock)	10 mg
DPBS	1×	10 mL
**Total**	**N/A**	**10 mL**

Mix well until BSA dissolves completely in DPBS.

Filter the BSA solution with a 0.2 μm filter.

Aliquots of 500 μL can be stored at −20°C.

If an aliquot is thawed, the BSA can be stored at 4°C for up to a month. Do not re-freeze the aliquot.

**Table:** Type I collagen-based coating for hPBECs and co-cultures

Reagent	Final concentration	Amount
PureCol	30 μg/mL	100 μL
Human Fibronectin	10 μg/mL	100 μL
BSA	10 μg/mL	100 μL
DPBS	1×	9.7 mL
**Total**	**N/A**	**10 mL**

Store at 4°C for up to 1 month. Coating can be re-used up to 3 times. For overnight coatings, use just once.

**Table:** Complete DMEM (for hLF growth and hMVEC isolation)

Reagent	Final concentration	Amount
DMEM	1×	500 mL
FBS	10% (vol/vol)	50 mL
P/S	1× (100 U/mL Penicillin and 100 μg/mL Streptomycin)	5 mL
**Total**	**N/A**	**555 mL**

Store at 4°C for up to 3 months.

**Table:** Complete KSFM medium (hPBECs growth medium)

Reagent	Final concentration	Amount
B/E aliquot	BPE: 25 μg/mL EGF: 0.2 ng/mL	∼90 μL
IP	1 μM	50 μL
KSFM medium + P/S	1×	50 mL
**Total**	**N/A**	**∼50 mL**

Beforehand: Prepare 50 mL aliquots of KSFM + 1% P/S (basic medium) and store at 4°C until use.

Complete KSFM medium can be stored at 4°C for up to 2 weeks.

**Table:** HPBECs freezing medium

Reagent	Final concentration	Amount
KSFM medium + P/S	1×	4.5 mL
B/E aliquot	BPE: 250 μg/mL EGF: 2 ng/mL	∼90 μL
DMSO	10%	0.5 mL
**Total**	**N/A**	**∼5 mL**

Prepare 5 cryovial aliquots of 1 mL each.

Freeze hPBECs cryovials at −80°C for up to 1 week. Move vials to liquid nitrogen tanks afterwards.

**Table:** 10× soft trypsin

Reagent	Final concentration	Amount
Difco Trypsin 1:250	0.3%	30 mg
EDTA	0.1%	10 mg
D-(+)- Glucose	1%	100 mg
DPBS	1×	10 mL
**Total**	**N/A**	**10 mL**

Filter the 10× soft trypsin solution with a 0.2 μm filter.

Divide the 10 mL of the 10× soft trypsin into 10 × 15 mL Falcon tubes (1 mL per tube).

Store at −20°C until use.

Thaw one 15 mL Falcon tube, and dilute the 1 mL of 10× Soft Trypsin with 9 mL of sterile DPBS to get 1× soft trypsin solution.

Use the 1× soft trypsin solution on the cells (1× soft trypsin final concentration is 0.03% Difco Trypsin, 0.01% EDTA, 0.1% Glucose in 1× DPBS).

Store at 4°C for up to 1 month.

**Table:** BEGM medium (hPBECs differentiation medium)

Reagent	Final concentration	Amount
2 × BEpiCGS	1×	2 × 5 mL
1 M HEPES buffer	12.5 mM	12.5 mL
BEpiCM medium	1/2 ×	500 mL
DMEM medium	1/2 ×	500 mL
**Total**	**N/A**	**∼1 L**

Mix DMEM and BEpiCM media at 1:1 ratio. Add all the supplements. Mix well.

Make 45 mL aliquots.

Store at −20°C until use. Store at 4°C for short periods of time.

**Table:** Other solutions

Name	Reagents, preparation and storage
Basal DMEM	Add 5 mL P/S (final concentration 1×: 100 U/mL Penicillin and 100 μg/mL Streptomycin) in 500 mL DMEM medium. Used for storing lung tissues prior to cell isolation. Store at 4°C for up to 3 months.
IP stock preparation for KSFM medium	Add 12,39 mg IP (final concentration 1 mM) into 50 mL KSFM medium with P/S. Filter the IP solution with a 0.2 μm filter. Make 2 mL aliquots: from these make 50 μL aliquots. Store at −20°C until use.
EGF stock preparation for KSFM medium	Add 2.5 μg EGF (final concentration 2.5 μg/mL) into 1 mL KSFM medium with P/S. Mix well until EGF dissolves completely in basic KSFM. Make 80 μL aliquots and store them at −20°C until use.
B/E aliquots: BPE and EGF aliquots preparation	Mix about 1.7 mL∗ of BPE and one 80 μL EGF aliquot together. ∗The provider supplies us with a vial containing 25 mg of BPE (in liquid form, usually around 1.7 mL). However, it is important to check the volume every time per vial as volume may vary. Mix BPE and EGF stock well. Divide the total volume (approx. 1.8 mL) into 20 Eppendorf tubes to make the B/E aliquots. Total volume is usually 80-90 μL per B/E aliquot, and final concentrations of BPE (25 μg/mL) and EGF (0.2 ng/mL). Store B/E aliquots at −20°C until use.
SBTI preparation	Add 220 mg SBTI (final concentration 1.1 mg/mL) into 200 mL KSFM medium with P/S. Filter the SBTI solution with a 0.2 μm filter. Aliquot 10 mL of SBTI solution into 15 mL Falcon tubes. Store at −20°C until use. Store at 4°C for short periods of time (up to 1 month).
EC-23 stock preparation	Dissolve 10 mg EC-23 (final concentration 5 mM) into 6.2 mL of pure EtOH. Make 5 μL aliquots. Store at −80°C for up to 6 months. Use a new aliquot every time when refreshing medium. In cell culture: make a 50 μM stock solution (1:100 dilution) by adding 495 μL of medium or DPBS into the 5 μL aliquots.
10× Collagenase type I solution	Dissolve 10 mg collagenase type I (final concentration 1 U/mL) into 10 mL sterile ddH_2_O. Store 10× solution at −20°C for up to 1 month. Make a 1× stock solution (1/10 of 10× stock solution into 9/10 of ddH_2_O) so final concentration is 0.1 U/mL. Prepare 1× stock solution fresh and use immediately. Discard after use.
Dispase I solution	Add 1 mL sterile ddH_2_O into the powder dispase (final concentration 0.8 U/mL) flask. Store at 4°C for up to 2 weeks.
MACS buffer	Add 0.1 g BSA (final concentration 0.1% wt/vol) into 100 mL DPBS. Filter with a 0.2 μm filter. Aliquot and store at −20°C until use. Store at 4°C for up to 2 weeks.
Glacial Acetic acid in sterile water	Add 57.47 μL (from 17.4 N stock) glacial acetic acid (final concentration 0.02 N) into 50 mL sterile ddH_2_O. Store at 4°C until it is finished.
HMVECs coating	Add 50 μL collagen R solution (0.4%) into 4 mL 0.02 N glacial acetic acid in sterile water Use once. Make fresh every time.

## Step-by-step method details

### Part 1: Isolation and culture of primary human lung cells

**Timing: 4–5 h (isolation)**

**Figure 1:**
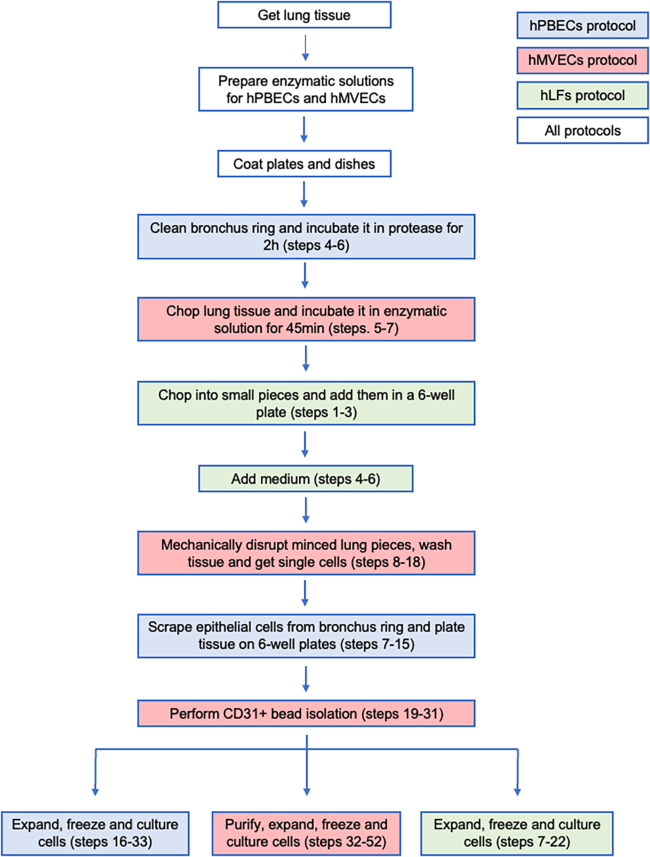
Optimized workflow for the isolation of hPBECs, hMVECs, and hLFs from human lung tissue Isolate all cell types on the same day (vertical chart). Timing of all tissue’s isolation: 5 h. In the future (bifurcations), expand and freeze cells at different paces.

Prior to obtaining the human lung tissue, it is mandatory to obtain approval from your local ethical committee. It is essential to follow to the optimized cell isolation and culture workflow outlined in Figure 1. In addition, ensure that all surgical instruments (scissors, tweezers, and scalpels) are readily available in stock and have been sterilized via autoclaving.

**Figure 2:**
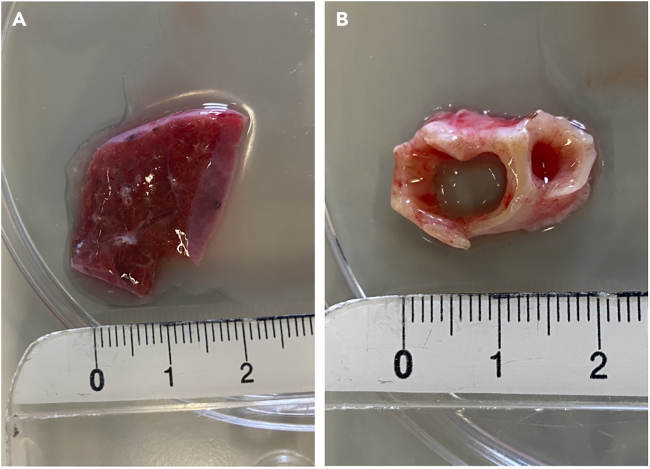
Tools and tissue needed for obtaining human lung primary cells (A) Picture of a piece of healthy human lung tissue, ruler for scale (cm). (B) Picture of a piece of healthy human bronchus ring, ruler for scale (cm).

#### Obtain human lung tissue from resection material

1. Collect lung tissue (Figure 2A) and a bronchus ring (Figure 2B) from resection material.
  ***Note:*** The protocol is applicable to human lung tissue irrespective of gender or age.
  a. Make sure the tissue is at least 2–3 cm^3^.
  b. Collect the tissues in Basal DMEM.
2. The tissue can be processed immediately or stored at 4°C in Basal DMEM up to 24 h.

***Note:*** Do not continue if the tissue is left ON without medium.

3. Divide the lung tissue into two pieces: 2/3 for the hMVECs and 1/3 for the hLFs.
4. Wash the lung piece in a 50 mL Falcon tube with DPBS to remove excess of blood, usually once or twice is sufficient. Agitate softly.

#### Isolation and culture of hPBECs

**Timing: 4 h**

Isolation of hPBECs

**Figure 3:**
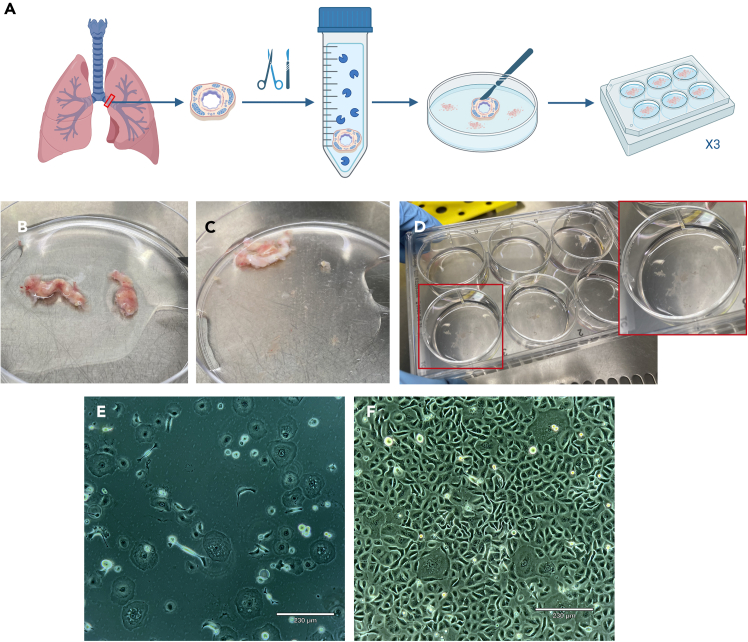
Isolation of hPBECs (A) Illustration showing the hPBECs isolation process. Illustration was made using BioRender.com. (B) The bronchus ring is cleaned of connective tissue, blood and mucus using sterile tweezers and scissors. Then it is cut open to improve the efficacy of the digestive solution. (C) After digestion, the bronchus ring becomes soft. (D) Once the bronchus ring is scraped, pieces of bronchus tissue are evenly distributed into the pre-coated 6-well plates. (E) HPBECs plated on 6-well plates after a couple days post isolation. Scale bar = 230 μm. (F) Healthy confluent hPBECs ready for freezing or plating on inserts. Scale bar = 230 μm.

The hPBECs isolation process is summarized in Figure 3A.

5. Get a 15 mL Falcon tube and prepare 0.18% Protease type XIV in 1× DPBS.
6. From this point on, work under sterile conditions in a cell culture hood.
7. Coat 3 × 6-well plates with co-culture coating at 37°C for 2 h. Add 1 mL of coating per well.
8. Get a human bronchus ring. Remove connective tissue, blood and mucus from the bronchus ring using sterile tweezers and scissors.
  a. Transfer the bronchus ring into a coated 10 cm Petri dish.
  b. Remove remaining blood, connective tissue and mucus with scissors and tweezers.
  c. Keep the cartilaginous white tissue (bronchus, Figure 3B).
9. Cut the ring open.

***Note:*** To keep surgical tools sterile and clean of tissue and blood, submerge them first in a 50 mL tube filled with DPBS. Then, submerge them in another 50 mL tube filled with 70% EtOH, and allow them air dry in the cell culture cabinet.

10. Incubate the bronchus ring in 0.18% protease XIV in DPBS at 37°C for 2 h.
  a. Use a water bath, not an incubator.
  b. Softly shake the tube every 20–30 min.

***Note:*** After digestion, the bronchus ring will become quite soft and gelatinous (Figure 3C).

11. Take the bronchus ring out of the protease solution and place it in a new and uncoated 10 cm dish with 10 mL 1× DPBS.

***Note:*** After the protease digestion, the bronchus ring will release some tissue into the protease solution. Collect this loose tissue by centrifuging it. Remove the solution, add DPBS and resuspend. Set it aside until step 14.

12. Scrape epithelial cells from the inner layer of the bronchus ring with a scalpel and tweezers.
13. Collect the loosened tissue in DPBS and add it to a new 50 mL Falcon tube.
14. Rinse the 10 cm Petri dish with 10 mL DPBS twice to collect the cells attached to the dish. Add these washes into the same 50 mL Falcon tube.

***Note:*** Add the DPBS containing the loose tissue from the bronchus ring of step 13.

15. Centrifuge at 300 g for 7 min.
16. Resuspend pellet in complete KSFM medium + GA-1000 (dilute 1:1000). Ensure to resuspend well so that the tissue is as loose as possible.
  a. For 3 × 6-well plates, use 36 mL of medium (2 mL/well).
17. Remove the coating from 3 × 6-well plates and wash them once with DPBS.
18. Divide cells and left over bronchus ring as homogeneously as possible over the 3 × 6-well plates (Figure 3D).
19. Change complete KSFM medium + GA-1000 after 24-48 h to remove the floating loose tissues and then change medium every other day. At that point, the first hPBECs will start to appear (Figure 3E). In about a week, hPBECs should be confluent (Figure 3F).

Trypsinizing hPBECs

20. Discard medium.
21. Wash once with DPBS.
  a. 10 cm dish: use 10 mL DPBS.
  b. 1 well of 6-well plate: use 2 mL DPBS.
22. Trypsinize cells using 1× soft trypsin.
  a. 10 cm dish: use 2 mL soft trypsin.
  b. 1 well of 6-well plate: use 1 mL soft trypsin.
23. Bring cells at 37ºC for 12-15 min.
24. Quench trypsin by adding double amount of SBTI.
25. Collect cells into a 15 or 50 mL Falcon tube.
26. Centrifuge at 400 g for 3 min.

Seeding hPBECs on inserts (as monocultures) at ALI

27. Prior: coat the apical side of insert membranes with co-culture coating at 37°C for at least 2 h (up to 24 h).
28. Discard supernatant.
29. Trypsinize hPBECs as described in the trypsinizing hPBECs section above.
30. Add 1 mL of BEGM medium and resuspend.
31. Count cells using Countess 3 Automated Cell Counter.
32. Wash inserts once with DPBS.
33. Seed hPBECs on inserts at a cell density of 1.0–1.5∗10^5^ cells/cm^2^.
34. Use BEGM medium with 1 nM EC-23 in both (apical and basolateral) chambers of the insert.

***Note:*** To improve hPBECs attachment to insert membranes, use 5 μM RI.

35. After 3-4 days of the hPBECs being submerged, put them at ALI.
  a. Discard medium of apical chambers to air-expose hPBECs.
  b. Add BEGM + 50 nM EC-23 + in the basolateral chamber.
    i. 12 mm inserts: 1 mL.
    ii. 6.5 mm inserts: 500 μL.

***Note:*** Ensure that hPBECs are completely confluent before putting them at ALI, otherwise they will not differentiate properly. For improved visualization of hPBECs under the microscope, remove the medium from the apical chamber.

36. Differentiate hPBECs at ALI for at least 14 days (up to a month).
37. Change medium 2–3 times a week.

#### Isolation and culture of hMVECs

Isolation of hMVECs

**Timing: 3 h**

**Figure 4:**
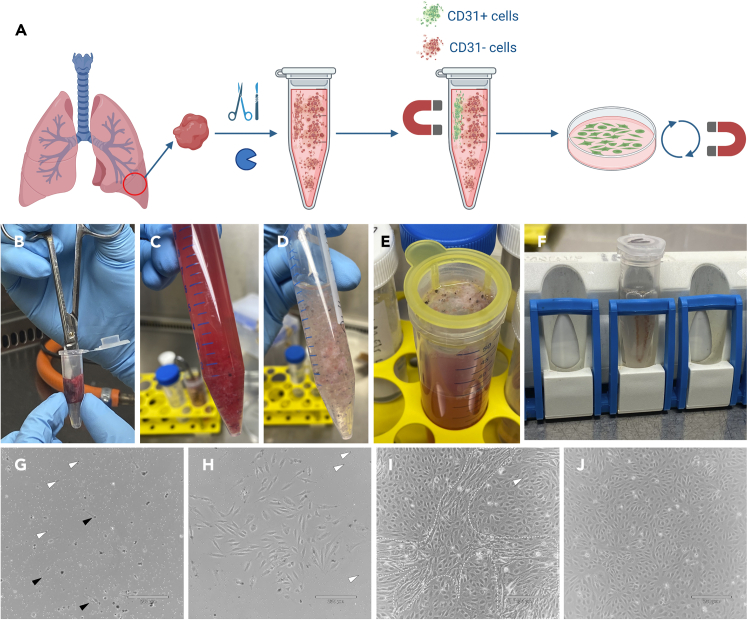
Isolation and purification of of hMVECs (A) Schematic illustration showing the hMVECs isolation process. Illustration was made using BioRender.com. (B) Piece of lung tissue was placed in an Eppendorf tube and minced with scissors for 5 min. (C) Minced lung tissue containing a significant amount of blood. (D) Clean minced lung tissue solution (without blood). (E) Cells were filtered from the lung tissue using a 100 μm cell strainer placed over a 50 mL Falcon tube. (F) Image of an Eppendorf tube placed in the magnetic rack, showing the attachment of CD31-dynabeads (brown) to the magnet. (G) Day 2 post isolation of hMVECs. The first endothelial cells can be spotted upon isolation (black arrowheads). (H) Day 7 post isolation of hMVECs. The first colonies of endothelial cells start forming. (I) Image of hMVECs containing a contaminating population of MSCs (inside the dotted white lines). Mesenchymal cells have a spindle-shaped morphology compared to the cobblestone morphology of endothelial cells. (J) Pure and healthy population of confluent hMVECs. Scale bars = 230 μm. White arrowheads = CD31 dynabeads.

The hPBECs isolation process is summarized in Figure 4A.

38. Prepare 1× collagenase type I solution.
39. Prepare dispase I solution.
40. Prepare 1× working enzymatic solution and put on ice until use. Use about 5 mL of the solution per 0.5 g of wet lung weight.
  a. For Example: for 1.5 g tissue, add 43 μL of 1× collagenase type I solution and 400 μL dispase solution into 15 mL DPBS + CaCl_2_ + MgCl_2_.
41. Coat one 6 cm dish with hMVECs coating at 37°C for at least 2 h. Add 2 mL of coating per 6 cm dish.
42. Transfer the lung tissue piece to an Eppendorf tube and finely mince it using scissors for a minimum of 5 min (Figure 4B).

***Note:*** This step is crucial for effective cell isolation. The finer the mincing process, the better the cell yield.

***Note:*** Wash the minced lung tissue with DPBS as needed until a clear cell suspension is obtained, because the presence of excessive blood may impede the effectiveness of the digestive enzymes. (Figures 4C and 4D).

***Note:*** To keep surgical tools sterile and clean of tissue and blood, submerge them first in a 50 mL tube filled with DPBS. Then, submerge them in another 50 mL tube filled with soap and water and then 70% EtOH, and allow them air dry in the cell culture cabinet.

43. Add the minced tissue into a 50 mL tube containing the 1× working solution of collagenase/dispase using a 5 mL pipette.
  a. Using a 5 mL pipette, draw up 0.5–1 mL of 1× working solution, and carefully dispense a portion of this volume into the Eppendorf tube containing the minced tissue. Draw up a small amount of the minced tissue and transfer it to the 50 mL tube. Repeat this process until all minced tissue has been transferred from the Eppendorf tube to the 50 mL tube.
44. Digest the lung tissue by placing the 50 mL tube into a 37°C water bath for 45 min.
  a. Gently shake the tube every 10-20 min to prevent lung pieces from pelleting.
45. Mechanically disrupt the minced lung pieces by pipetting initially with a 5 mL pipette, followed by a cut P1000 tip attached to a 5 mL or 10 mL pipette.
  a. Use first a 5 mL pipette and resuspend the minced lung solution 15–20 times. No need to be gentle. Make sure that the minced pieces pass smoothly through the pipette without getting stuck.
  b. Cut the tip of P1000 tip and attach it to a 5 mL or 10 mL pipette. Resuspend the minced lung solution 15-20 times. No need to be gentle either. Minced pieces have to be able to go through the pipette tip without much problem.
46. Vortex the tube 3 × 5 s each.
47. Use a dissection microscope to confirm the presence of floating cells dispersed around the tissue.
  a. Put the Falcon tube perpendicular to the objective of the bright-field light microscope. Use 2× objective and focus.
  b. Look for large, transparent cells. If there are a few or no cells: repeat step 45 to 47.
48. Quench the 1× working solution with an equal volume of complete DMEM.
49. Draw the mixture up and down several times using a 5 mL pipette.
50. Filter cells over a 100 μm strainer (Figure 4E).
  a. Place a 100 μm cell strainer on a new 50 mL tube. If an excessive amount of tissue obstructs the strainer, use two strainers. Pipette into the center the strainer to disrupt the surface tension. Be careful not to overflow.
  b. Use a 5 mL syringe plunger to help the liquid go through the strainer.
    i. Be gentle, you want to avoid rupturing the strainer.
    ii. Gently press on the minced tissues and draw circles in the strainer.
51. Wash the cell strainer with 10 mL of complete DMEM.
  a. Use the syringe plunger again until all liquid has passed through the strainer.
  b. Discard the strainer with the minced tissue.
52. Centrifuge the 50 mL tube at 300g for 10 min.
53. Remove supernatant and resuspend the cell pellet in 10 mL of complete DMEM. Transfer the solution into a 15 mL Falcon tube.
54. Centrifuge the tube at 300g for 10 min.
55. Remove supernatant and resuspend the cell pellet in 1 mL of magnetic-activated cell sorting (MACS) buffer. Place cells on ice.

CD31^+^ bead isolation

56. Homogeneously mix the CD31 Dynabeads.
57. Take 5 μL of Dynabeads in a 1.5 mL Eppendorf tube and add 1 mL of MACS buffer to wash the beads. Mix well.
58. Add the tube to the Eppendorf magnetic rack (Figure 4F). Wait 20–30 s until all beads are attached to the magnet.
59. Discard the supernatant by pipetting it out (or use a vacuum very gently with a P200 tip attached to avoid touching the beads), and add the cell solution from step 55.
60. Incubate cells with the CD31 Dynabeads for 30 min at 4°C under gentle rotation.
61. Place the tube again in the Eppendorf magnetic rack until the Dynabeads are attached to the magnet (about 20–30 s).
62. Discard the supernatant by pipetting it out (or use a vacuum very gently with a P200 tip attached).
63. Remove the Eppendorf tube from the magnet and wash the bead-bound cells by adding 1 mL of MACS buffer. Resuspend until the solution is homogeneous.
64. Repeat Steps 61 to 63 up to four times (4 washes in total).
65. Remove the coating from the 6 cm dish, wash it once with DPBS, and add 3 mL of EGM-2MV medium into the dish.
66. Resuspend the cells bound to the CD31 Dynabeads in 1 mL of EGM-2MV medium, then transfer the entire solution into the 6 cm dish (total volume 4 mL). Make sure the cells are spread evenly on the dish. Bring the dish back to the incubator.
67. After 48 h, refresh medium.
  a. Slowly aspirate the medium from the well at a rate of 1 mL per 4–5 s. The cells are still partially attached to the dish due to the Dynabeads (Figure 4G).
  b. Slowly add 4 mL of fresh EGM-2MV medium.
68. After a few days, the first endothelial cell colonies will start to form (Figure 4H).

Further purification and expansion.

Contamination with other cell types (i.e. mesenchymal-like cells, MSCs) is usually observed in the isolation of hMVECs (Figure 4I). Therefore, conduct a maximum of four CD31^+^ Dynabeads isolations in total. Allow them to grow until they reach confluency to facilitate better observation of any MSC-like cells. While expanding the cell population to increase cell count, we can also purify them.

69. Coat one (in the second CD31^+^ bead isolation), three (in the third CD31^+^ bead isolation), or 6–8 (in the fourth CD31^+^ bead isolation) x 10 cm dishes with hMVEC coating for at least 2 h at 37°C. Add 4 mL of coating per 10 cm dish.
  a. At the second or third isolation (depending on the purity of the endothelial cell population), it is advisable to coat one well of a 6-well plate or a 6 cm dish to assess the purity of endothelial cells via FACS sorting (section below).
70. Discard the medium from the 6 cm dish and wash the cells once with DPBS.
71. Add 1 mL of TE buffer into the 6 cm dish. Incubate for at least 3 min until the endothelial cells detach.

***Note:*** It is common to encounter numerous MSCs in the initial purification round. Simply add the TE buffer, and endothelial cells detach more rapidly than MSCs. Thoroughly wash the dish with TE buffer using a P1000 pipette to detach and collect the endothelial cell, while leaving as many MSCs as possible behind.

72. Wash the dish with 2 mL of 10% FBS in DPBS and collect the detached cells into a 15 mL Falcon tube.
73. If needed, wash the dish one or two additional times until all endothelial cells are collected.
74. Centrifuge cells at 400 g for 3 min.
75. Discard supernatant and resuspend the cell pellet in 1 mL of MACS buffer.
76. Repeat the bead isolation process from step 55 to step 67 using 3–5 μL CD31-Dynabeads (depending on the number of plates available). Incubate cells at 4°C for 20 min with gentle rotation.
77. Discard coating from the 10 cm dish(es) and wash it once with DPBS.
78. Add the cells evenly to each dish containing 10 mL of EGM-2MV medium per 10 cm dish.
79. Cell purity can be either check by IF or FACS (Figure 4J).

Trypsinizing hMVECs.

80. Discard medium.
81. Wash once with DPBS.
  a. 10 cm dish: use 10 mL DPBS.
  b. 1 well of 6-well plate: use 2 mL DPBS.
82. Trypsinize cells using TE buffer.
  a. 10 cm dish: use 2 mL TE buffer.
  b. 1 well of 6-well plate: use 1 mL TE buffer.
83. Put cells at 37°C for 3-5 min.
84. Quench TE buffer by adding double amount of 10% FBS in DPBS.
85. Collect cells into a 15 or 50 mL tube.
86. Centrifuge at 400 g for 3 min.
87. Aspirate the supernatant from the cell pellet and resuspend cells in 1 mL of complete EGM-2MV medium.
88. Count cells using Countess 3 Automated Cell Counter.
89. Plate cells on freshly coated inserts o plates.

#### Isolation and culture of hLFs

**Timing: 1 h**

Isolation of hLFs.

**Figure 5:**
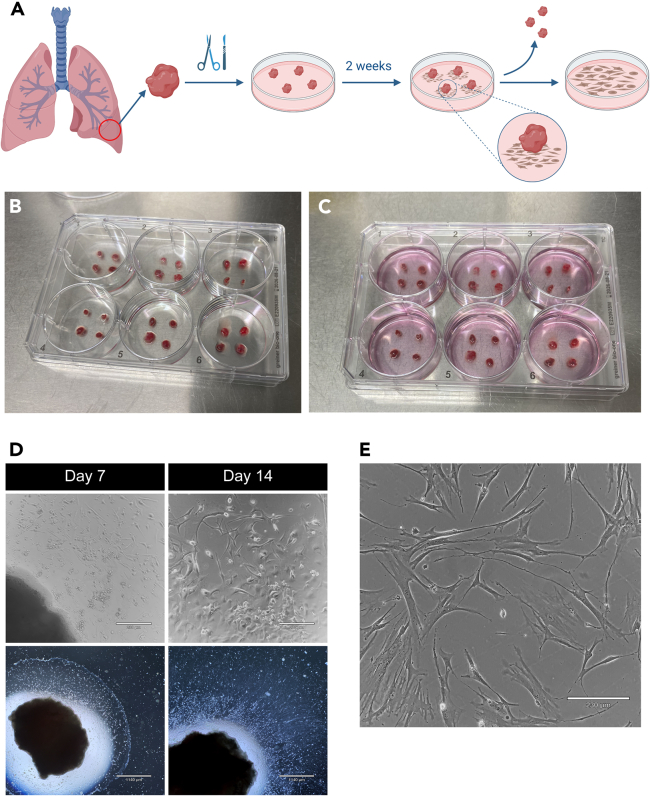
Isolation of hLFs (A) Schematic illustration showing the hLFs isolation process. Illustration was made using BioRender.com. (B) Image showing lung tissues chopped into 0.3 cm^2^ pieces and added in a 6-well plate without medium. The plate was then placed in the incubator to improve lung pieces attachment to the plate. (C) Image showing lung tissue pieces still attached to the plate after medium addition. This step is crucial to allow hLFs to migrate out of the tissue and to attach onto the plate. (D) Images on Day 7 and 14 post isolation showing hLFs attached on the plate. Black spots in bottom images are the lung pieces. Scale bars = 230 μm (top pictures). Scale bars = 1140 μm (bottom pictures). (E) Normal and evenly distributed hLFs. Scale bar = 230 μm.

The hLFs isolation process is summarized in Figure 5A.

90. Take a piece of lung tissue and place it in a 10 cm dish.
91. Remove any abnormal appearing parts of the lung, such as black spots, if necessary.
92. Cut 0.3 cm^2^ pieces of lung tissue with scissors and tweezers.
93. Add 3–4 pieces per well of a 1× 6-well plate (Figure 5B). Without adding medium yet, allow the pieces to adhere onto the surface of the well by keeping the plate in a humidified incubator at 37°C for 20 min.

***Note:*** Lung tissue floats when medium is added. Therefore, it is crucial to do this step so that the lung pieces stay attached on the plate. This way, fibroblasts can migrate out of the tissue and adhere to the plate.

94. Use a P1000 pipette to add very carefully 1 mL of complete DMEM medium with GA-1000 (dilute 1:1000) per well (Figure 5C).
95. Place the 6-well plate back in the incubator.
96. The 6-well plate should remain undisturbed in the incubator for a minimum of 48 h, without any movement or medium change to facilitate further adhesion of the lung pieces.

***Note:*** If the medium turns yellow in 24 h, consider checking for a potential bacterial infection (troubleshooting).

97. Refresh each well with 1 mL of medium every other day.
  a. Be careful while changing medium not to detach the lung pieces from the well.
  b. Discard medium and add fresh medium using a P1000 pipette.
98. After 1 week of culture, the first fibroblasts will start appearing at the base of the lung pieces (Figure 5D).
99. Allow the tissues to remain in culture for a total of 2 weeks to ensure more fibroblasts settle on the plate (Figure 5D).
  a. Usually, two weeks suffice to ensure enough number of fibroblasts surrounding the lung piece.
100. Once an adequate number of fibroblasts are present, transfer the tissues to a T-25 flask containing 10 mL of complete DMEM with GA-1000. Place the flask vertically and in the incubator.

***Note:*** This step serves as a precautionary measure in case any issues arise later in the protocol, ensuring that we still have the tissues available to obtain additional fibroblasts if needed. Otherwise, the lung pieces can be discarded immediately.

101. Split the adhered fibroblasts into a new 6-well plate to homogeneously distribute them and to obtain a higher cell yield (Figure 5E). Use complete DMEM supplemented with 1× GA-1000.

Trypsinizing hLFs

102. Discard medium.
103. Wash once with DPBS.
  a. 10 cm dish: use 10 mL DPBS.
  b. 1 well of 6-well plate: use 2 mL DPBS.
104. Trypsinize cells using TE buffer.
  a. 10 cm dish: use 2 mL TE buffer.
  b. 1 well of 6-well plate: use 1 mL TE buffer.
105. Put cells at 37°C for 3–5 min.
106. Quench TE buffer by adding double amount of complete DMEM.
107. Collect cells into a 15 or 50 mL Falcon tube.
108. Centrifuge at 400 g for 3 min.
109. Discard supernatant from the cell pellet and resuspend cells in 1 mL of complete DMEM.
110. Count cells using Countess 3 Automated Cell Counter.

### Part 2: Combined cultures of primary cells

#### hPBECs and hMVECs co-culture setup

**Timing: 1 week**

**Figure 6:**
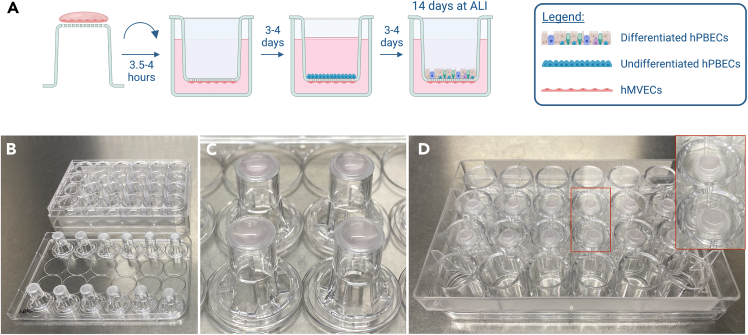
Establishing a hPBECs-hMVECs airway co-culture model (A) Schematic illustration of the entire co-culturing process. Images were made using BioRender.com. (B) After discarding the coating solution, inserts were flipped upside-down and allowed to dry for 2–3 h in a sterile cell culture cabinet. (C) Droplets of hMVEC suspension were added on each insert. (D) Upside-down plate was added on top of the inserts. Droplets covered the entire surface of the insert membrane without overflowing. HMVECs were allowed to attach for 3.5–4 h at 37°C. The plate was then flipped back upright to continue with the protocol.

This protocol offers comprehensive guidance on establishing co-cultures of human lung primary hPBECs and hMVECs on opposite sides of cell culture insert membranes. It is crucial to execute all steps within a sterile cell culture cabinet. A summary of the hPBECs-hMVECs co-culture process is shown in Figure 6A.

111. Coat the basolateral side of 12 or 6.5 mm cell culture insert membranes with co-culture coating overnight (ON).
  a. 12 mm inserts: 1 mL coating/well.
  b. 6.5 mm inserts: 500 μL coating/well.
112. Next day, discard the coating solution.

**CRITICAL:** Flip the inserts upside-down and allow them to rest on the lid without the plate. Let the coating air dry for 2-3 h in the cell culture cabinet (Figure 6B) (troubleshooting).

**Figure 7:**
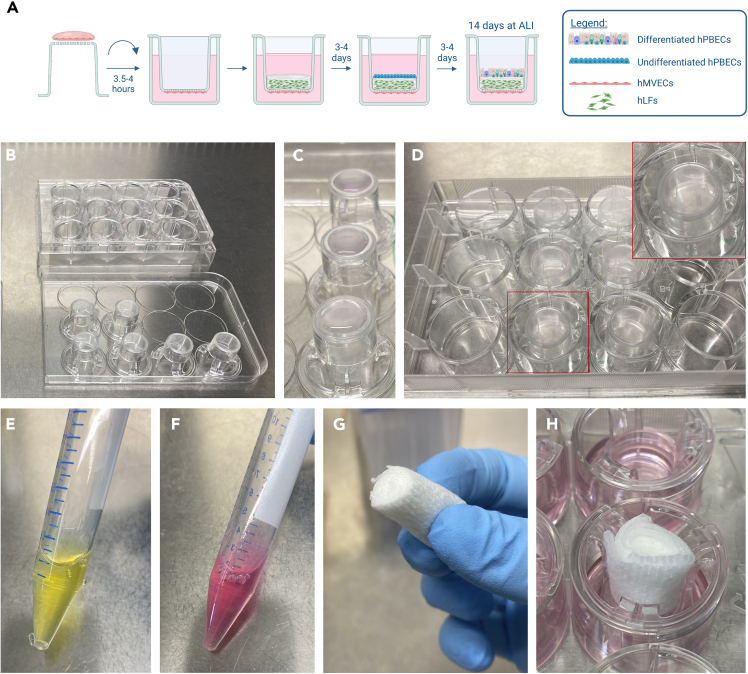
Establishment of a three-dimensional *in vitro* airway tri-culture model (A) Schematic illustration of the setting up process. Images were made using BioRender.com. (B) After discarding the coating solution, inserts were flipped upside-down and allowed to dry for 2–3 h in a sterile cell culture cabinet. (C) Droplets of hMVEC suspension were added on each insert. (D) Upside-down plate was added on top of the inserts. Droplets covered the entire surface of the insert membrane without overflowing. HMVECs were allowed to attach for 3.5–4 h at 37ºC. The plate was then flipped back upright to continue with the protocol. (E) Collagen solution with 10× MEM. Solution remains yellow due to its acidic pH. (F) Collagen solution with 10× MEM and RB. Solution turned pink upon addition of RB, indicating a neutral pH, and readiness for rapid polymerization at 37ºC. G-H) Check troubleshooting section. (G) Sterile gauze pad was cut and rolled up to fit insert. (H) Collagen gels were gently flattened using the rolled up sterile gauze pads.

113. Do not wash the inserts with DPBS.
114. Seed hMVECs directly on the basolateral side of insert membranes at a cell density of 2∗10^5^ cells/cm^2^ in complete EGM-2MV medium.
  a. Check trypsinizing hMVECs section (supplementary information).
  b. 6.5 mm inserts (Figure 6C): 6.7∗10^4^ cells in 50 μL EGM-2MV (per Costar insert) or in 40 μL EGM-2MV (per Celltreat insert).
  c. 12 mm inserts (Figure 7C): 2∗10^5^ cells in 100 μL EGM-2MV (per insert).
115. Carefully place the plate back onto the inserts.
  a. Make sure the plate perfectly sits on the lid and that the medium covers the whole surface of the insert without overflowing.
  b. Bring the plate (upside down) back to the incubator to let endothelial cells attach to the insert membrane (Figure 6D).
116. Allow hMVECs to adhere to the membrane for 3.5–4 h at 37°C.
117. Flip the plate back, remove any residual supernatant droplets, and add EGM-2MV medium only in the basolateral chamber.
  a. Leave the apical chamber without medium and coating.
  b. Return the plates to the incubator.
  c. 6.5 mm inserts: add 500 μL EGM-2MV medium.
  d. 12 mm inserts: add EGM-2MV 1 mL medium.

***Note:*** Some hMVECs may begin to adhere to the bottom of the well. If there is an excessive number of cells adhering, consider replacing the insert plate.

118. After 3–4 days (up to a week), seed hPBECs on the apical side of insert membranes at a cell density of 1.5∗10^5^ cells/cm^2^ in BEGM with 5 μM Rock Inhibitor (RI) and 1 nM EC-23.
  a. Coat the apical side of insert membranes with co-culture coating for at least 2 h at 37°C. Add enough coating to ensure complete coverage of the entire membrane surface.
  b. Check trypsinizing hPBECs section (supplementary information).
  c. Add 1 mL BEGM and count cells with Countess 3 Automated Cell Counter.
  d. Discard the coating solution and rinse it once with DPBS.
  e. For 6.5 mm inserts: add 5∗10^4^ cells in 150 μL BEGM with RI and EC-23 (per insert).
  f. For 12 mm inserts: add 1.5∗10^5^ cells in 400 μL BEGM with RI and EC-23 (per insert).
  g. Change the medium of the basolateral chambers for co-culture medium BEGM:EGM-2MV (2:1 ratio).

***Note:*** Once hPBECs are added on the inserts, make sure that the basolateral chamber is filled with BEGM:EGM-2MV (2:1 ratio) co-culture medium while the hPBECs apical chamber is only filled with BEGM.

***Note:*** RI improves hPBECs attachment and survival during the seeding procedure.^13^

119. After 3–4 days of the hPBECs being submerged, put them at ALI.
  a. Discard medium of the apical chamber to air-expose the hPBECs.
  b. Add co-culture medium BEGM:EGM-2MV (2:1 ratio) with 50 nM EC-23 in the basolateral chamber.
  c. For 6.5 mm inserts: add 500 μL co-culture medium per insert.
  d. For 12 mm inserts: add 1 mL co-culture medium per insert.

***Note:*** Ensure that hPBECs reach full confluency before putting them at ALI, as inadequate confluency may impede proper differentiation and lead to cell death. To facilitate a clearer assessment of hPBECs confluency under the microscope, remove the medium from the apical chamber of the inserts.

120. Differentiate hPBECs at ALI for 14 days (up to 1 month). Optionally, differentiate hPBECs at ALI in the Simple-Flow device for 14 days.
  a. Change BEGM:EGM-2MV (2:1 ratio) co-culture medium with 50 nM EC-23 three times a week.
  b. Each time co-culture medium is replaced, add DPBS + CaCl_2_ + MgCl_2_ in the apical chamber to wash mucus from hPBECs. Place the plate back in the incubator for 5 min. Then, discard DPBS.
  c. Measure trans-epithelial electrical resistance (TEER) at least on Days 0, 7 and 14 of ALI. To measure TEER: add DPBS + CaCl_2_ + MgCl_2_ in the apical chamber and place the plate in the incubator for 5 min. Then, measure TEER and discard DPBS^12^ (see section expected outcomes).

***Note:*** HMVECs do not thrive when kept confluent for extended culture periods. Therefore, the longer hPBECs differentiate, the hMVECs may start to loose integrity.

***Note:*** Co-culture medium induces more hPBECs mucus secretion than BEGM alone. If co-cultures produce a lot of mucus, the apical side of the cultures can be washed with DPBS + CaCl_2_ + MgCl_2_ every time cell culture media is changed.

***Note:*** While measuring TEER and washing mucus, it is crucial to use DPBS + CaCl_2_ + MgCl_2_ to avoid disrupting tight junctions.^14^^,^^15^^,^^16^ DPBS washes alone should be prevented.

121. After 14 days of hPBECs differentiating at ALI, the co-cultures need to be processed for downstream analysis, such as qPCR or immunostaining.
  ***Note:*** Cells may be harvested and stored as cell pellet at −80°C for the isolation of RNA, or fixed for immunofluorescence (IF) staining, as described below.
  a. Discard medium from basolateral chamber.
  b. Wash co-culture once with DPBS + CaCl_2_ + MgCl_2_ (apical and basolateral chambers).
  c. Fix co-cultures with 4% paraformaldehyde (PFA) at room temperature (RT) for 10 min.
  d. Wash co-cultures 3 times with DPBS to remove residual PFA.
  e. Store co-cultures in DPBS at 4°C up to 1 week.

#### hPBECs, hMVECs tri-culture setup

**Timing: 1 week**

This protocol describes the necessary steps to establish a three-dimensional tri-culture model of the human airway. It consists of hMVECs plated on the basolateral side of insert membranes, hLFs embedded in a type I collagen gel, with hPBECs placed on top. All steps should be carried out in a sterile cell culture cabinet. A summary of the tri-culture process is shown in Figure 7A.

122. Follow steps 111-116 of hPBECs and hMVECs co-culture setup section above*.* Use 12.5 mm Costar cell culture inserts (Figures 7B–7D).
123. While endothelial cells attach on insert membranes, prepare collagen solution in the following order and ratios: Cellmatrix type I-A: 10× MEM: Reconstitution Buffer (RB) (8:1:1). Final hydrogel concentration 2.4 mg/mL. Per 12 mm insert, use 200 μL of collagen solution. That is the minimum quantity of collagen required to cover the entire surface of a 12 mm insert.
  a. In a 15 mL Falcon tube, add the Cellmatrix type I-A collagen solution. Use a 5 mL pipette or P1000 tips.
  b. Add the 10× MEM with a P100 or P200 pipette until the solution is homogeneously yellow (Figure 7E).
  c. Add the RB with a P100 or P200 pipette until the solution is homogeneously pink (Figure 7F).
  d. Place the collagen solution on ice and allow it to rest for approximately 30 min.

***Note:*** Keep all components on ice to reduce collagen polymerization, especially when the components are already mixed.

***Note:*** Once the 3 components are thoroughly mixed, the solution should appear pink, indicating that pH is near 7. Neutral pH and temperatures ranging from 25 to 37°C are essential for the rapid polymerization of type I collagen gels in cell culture applications^17^^,^^18^ (Figures 7E and 7F). Therefore, to reduce collagen polymerization, the solution on ice.

***Note:*** Collagen solution is highly viscous. Thus, it is recommended to prepare 1.5–2 times more than needed, as a portion tends to adhere to pipette tips. Cold pipette tips prevent collagen adhering to the tips, thus store them in a freezer.

***Note:*** Be cautious not to introduce air bubbles into the collagen solution while pipetting. A helpful tip is to use a lower volume than the actual collagen solution to resuspend. For instance, if you have a total volume of 1 mL collagen solution, readjust the pipette and resuspend using 700 μL.

124. Trypsinize hLFs and mix them with the collagen solution.
  a. Trypsinize hLFs with Trypsin-EDTA (TE) buffer for 3–5 min at 37°C and quench the solution with complete DMEM. For further details check the trypsinizing hLFs section (supplementary information).
  b. Count cells with Countess 3 Automated Cell Counter or Neubauer chamber.
  c. Use 5∗10^3^ hLFs embedded in 200 μL collagen solution per 12 mm insert.
  d. Obtain the necessary amount of hLFs into a new 15 mL tube, centrifuge the cells at 400 g for 3 min, and discard the supernatant.
    ***Note:*** Ensure that the volume of the collagen solution is at least 50 times more than the hLFs resuspended solution.
  e. Add the necessary amount of collagen solution into the 15 mL tube containing the hLFs, and resuspend carefully without forming bubbles. Ensure to mix the cells homogeneously with the collagen solution. Put the samples on ice.
125. After allowing the hMVECs to attach for 3.5–4 h, get the plate from the incubator, flip it back upright, remove the residual supernatant from the droplets, and add 1 mL EGM-2MV medium in the basolateral chamber.
126. Add 200 μL of hLF-collagen solution in the apical chamber using a P200 pipette. If needed, gently agitate the plate to ensure uniform distribution of the solution within the insert.
127. Allow the collagen gels polymerize at 37°C for 60 min.
128. Once the gels are solidified, add 500 μL EGM-2MV medium in the apical chamber covering the gel.
129. Allow the hMVEC-hLF co-cultures to sit in the incubator for 3-4 days.

***Note:*** Instead of plating hPBECs immediately after collagen gelation, waiting a couple days results in hPBECs attaching more effectively on top these gels. HPBECs appear to attach better due to hLFs rearranging the collagen matrix, which stiffens the collagen matrix,^19^ therefore improving cell attachment.^20^^,^^21^

130. After 3-4 days, seed hPBECs on top of collagen gels at a cell density of 2.5∗10^5^ cells/cm^2^. Use 500 μL of BEGM:EGM-2MV (2:1 ratio) co-culture medium with 5 μM RI and 1 nM EC-23 per insert.
  a. Check trypsinizing hPBECs section (supplementary information).
  b. Add 1 mL BEGM and count cells with Countess 3 Automated Cell Counter.
  c. Discard medium from the apical chambers.
  d. Add 2.5∗10^5^ cells in 500 μL BEGM:EGM-2MV (2:1 ratio) with RI and EC-23 (per insert).

***Note:*** HPBECs do not adhere surface of collagen gels as well as they do to collagen-coated 10 cm dishes. Also, in the hPBECs-hMVECs co-culture, we use BEGM medium alone, as there are no other cell types present in the apical chamber. However, in the tri-culture model, hLFs, which thrive better in media containing FBS, are also present in the apical chamber. Hence, in the tri-culture model, we opted for co-culture medium during hPBECs seeding. For these reasons, hPBECs were seeded at higher densities in the tri-culture model, to aid them in achieving confluency more rapidly.

131. Replace EGM-2MV medium in the basolateral chamber for BEGM:EGM-2MV (2:1 ratio) co-culture medium with 1 nM EC-23.

***Note:*** After adding hPBECs to the hMVEC-hLFs co-cultures, ensure to switch to BEGM:EGM-2MV (2:1 ratio) co-culture medium in both the apical and basolateral chambers.

132. After 3-4 days of hPBECs being submerged, put them ALI.
  a. Discard medium from apical chambers to air-expose hPBECs.
  b. Add 1 mL of BEGM:EGM-2MV (2:1 ratio) co-culture medium with 50 nM EC-23 in the basolateral chamber.

***Note:*** Ensure that hPBECs reach full confluency before putting them at ALI, as inadequate confluency may impede proper differentiation and lead to cell death. To facilitate a clearer assessment of hPBECs confluency under the microscope, remove the medium from the apical chamber of the inserts.

133. Differentiate hPBECs at ALI for 14 days (troubleshooting).
  a. Change BEGM:EGM-2MV (2:1 ratio) co-culture medium with 50 nM EC-23 three times a week.
  b. Each time co-culture medium is replaced, add 500 μL of DPBS + CaCl_2_ + MgCl_2_ in the apical chamber to wash mucus from hPBECs. Place the plate back in the incubator for 5 min. Then, discard DPBS.
  c. Measure TEER at least on Days 0, 7 and 14 of ALI. To measure TEER: add 500 μL of DPBS + CaCl_2_ + MgCl_2_ in the apical chamber, and place the plate in the incubator for 5 min. Then, measure TEER and discard DPBS.

***Note:*** While measuring TEER and washing mucus, it is crucial to use DPBS + CaCl_2_ + MgCl_2_ to avoid disrupting hPBECs tight junctions.^14^^,^^15^^,^^16^ DPBS washes alone should be prevented.

***Note:*** Co-culture medium induces more mucus formation on hPBECs compared to BEGM alone.

134. After 14 days of hPBECs differentiating at ALI, process the cultures for downstream analysis.
  a. Discard medium from basolateral chamber.
  b. Wash tri-culture once with DPBS + CaCl_2_ + MgCl_2_ (apical and basolateral chambers).
  c. Fix co-cultures with 4% PFA at RT for 1 h.
  d. Wash co-cultures 3 × 10 min with DPBS to remove residual PFA.
  e. Store co-cultures in DPBS at 4°C up to 1 week.

### Part 3: 3D printing and post-processing of simple-flow device

**Timing: 11 h**

Below, we outline the steps necessary for the successful 3D-printing and post-processing of the Simple-Flow device. For further information regarding the Simple-Flow device, check here.^12^

135. Open file “Simple-Flow.form” with PreForm software (Formlabs) or “Simple-Flow.stl”.

***Note:*** For users without access to Formlabs technology, we have also included the Simple-Flow stl files. Both plates undergo identical printing and post-processing procedures. We advise using a resin that is biocompatible, such as dental resins, in order to improve cells viability.

**Figure 8:**
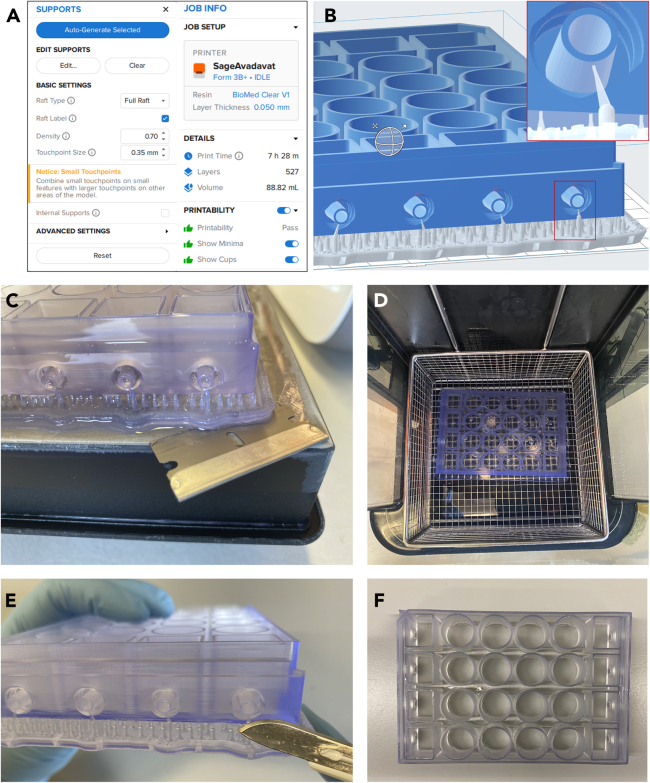
3D-printing and post processing of simple-flow device (A and B) Capture screenshot of the PreForm software interface showing (A) the specifications for the 3D printing of Simple-Flow, (B) highlighting the requirement of just one external support for printing the internal male Luer lock connectors. (C) A razor blade is used to carefully separate the 3D-printed device from the platform. (D) 3D-printed plate is washed twice with 100% IPA in an upward position in the Form Wash. (E) External supports are carefully cut with a sharp scalpel. (F) Top view of post-cured Simple-Flow, which is ready to use for cell culturing applications.

136. On the Preform Software, ensure the following printing specifications are met (Figure 8A):
  a. On the top left of the software interface, click on the “Supports” button and ensure that the density is set at 0.7 and touching point size at 0.35 mm.
  b. On the top right, make sure that the resin used is BioMed Clear, Tank V2.1 and the layer thickness is 0.05 mm.
  c. Ensure that each Luer connector has one support, and that there are no supports inside their hole (Figure 8B).
  d. Warnings on minima supports (red patches) can be ignored.
  e. Press the orange button, and press “Upload Job”.
137. Send file to the 3D printer, and follow the instructions on the printer.
138. Once the 3D printing process has finished, close the cap of the resin cartilage, and detach the platform (containing the printed plate) from the 3D printer.
139. Place the platform at −20°C for 15–20 min to help detaching the plate from the platform.
140. Using a razor blade, carefully detach the 3D-printed plate from the platform (Figure 8C).
  a. Starting in one corner, carefully insert and move the razor blade between the plate and the platform until lifting one side of the plate.
  b. Lift the plate cautiously.

***Note:*** Be careful not to scratch the platform.

***Note:*** Ensure to replace the razor blade every 3–4 prints. The blade gradually loses its sharpness, and it becomes more difficult to detach the plate. If you encounter difficulties in separating the plate from the platform, replace the blade with a new one.

141. Wash off the excess of uncured resin with 100% Isopropanol (IPA, Figure 8D) according to manufacturer’s protocol for BioMed Clear resin (https://support.formlabs.com). Briefly:
  a. Fill the Form Wash with enough clean IPA to cover all the 3D printed plate.
  b. Place the 3D-printed plate upwards into the metal grid basket of the Form Wash.
  c. Wash for 15 min.
  d. Meanwhile, clean the platform with clean IPA and place it back to the 3D printer.
  e. Replace the used IPA and add fresh one into the Form Wash.
  f. Wash plate again for 5 min.

***Note:*** Pour the used IPA into a transparent beaker, cover it with aluminum foil and allow the uncured resin to cure under sun light. After a few days, filter the IPA using paper filter and reserve it for future washes. Discard IPA when the resin concentration of resin becomes excessive or when it loses its transparency.

142. Allow the IPA dry from the 3D-printed plate. Use pressurized air to remove the excess of IPA from Luer connector holes and help the plate dry.
143. Cut out the supports carefully with a scalpel (Figure 8E).
  a. Cut out first the Luer connectors supports.
  b. Cut out the outer supports around the plate.
  c. Dethatch the inner supports carefully with your hands.
  d. Remove the remaining supports from the plate using both your hands and a scalpel, ensuring there are no residues left in the Luer connectors. If any remains are still found, carefully remove them using tweezers and a scalpel.
144. Enhance the rigidity and biocompatibility of the resin on the plate by subjecting it to additional UV light and heat treatment. Use the Form Cure according to manufacturer’s guidelines for BioMed Clear resin to achieve optimal results (https://support.formlabs.com). Briefly:
  a. Place the 3D-printed plate upwards into the Form Cure.
  b. Set the following settings:
    i. Curing time: 60 min.
    ii. Curing temperature: 60°C.
145. The Simple-Flow platform is now ready, store the plate at RT in a dark until further use (Figure 8F).

### Part 4: Setting up the simple-flow device with human airway co-cultures

**Timing: 2 h**

This protocol describes the necessary steps to set up the Simple-Flow device to facilitate the exposure of human lung co-cultures on cell culture inserts to a flow medium.

**Figure 9:**
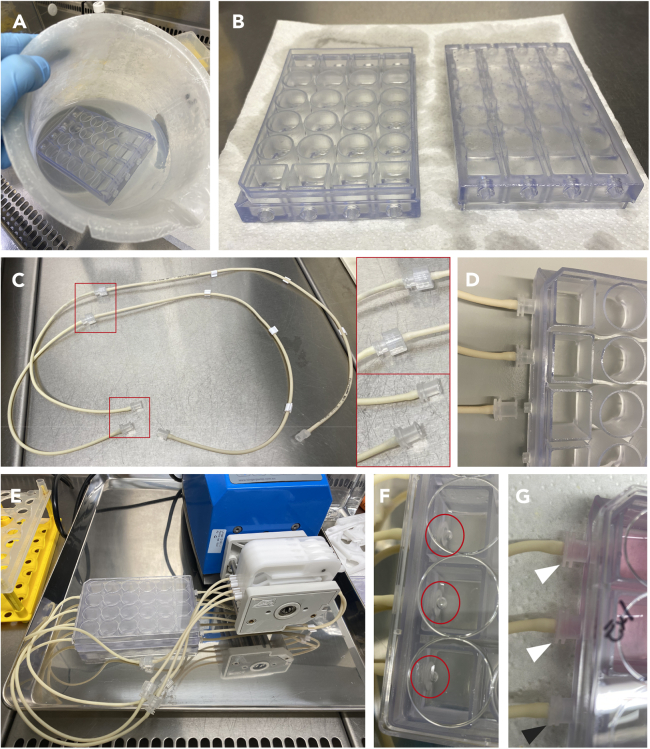
Preparing the simple-flow fluidic device for co-cultures on inserts (A) Simple-Flow plates were submerged in 70% IPA for 5-10 s to sterilize them. (B) Excess of IPA was removed with paper towels and Simple-Flow was air-dried in a cell culture cabinet. (C) Extension tubing was cut and connected to the three-stop tubing using male and female Luer lock connectors. Red squares show zoom in images of the connectors. (D) Tubing was connected to the 3D-printed plate using female Luer lock connectors. (E) Tubing was attached to the cassettes, which were then connected to the pump cylinder and positioned over the rollers. (F) Inspect the Simple-Flow plate’s holes, where Luer connectors are affixed, for the presence of bubbles to confirm that liquid flows through the tubing. Red circles indicate the formation of bubbles. (G) To ensure the flow of cell culture medium through the setup and tubing, the female Luer lock connectors were monitored until they turned pink. White arrowheads = connectors with medium. Black arrowheads = connector without medium.

146. Bring the following materials into a sterile cell culture cabinet:
  a. 3D-printed plates.
  b. About 2 L of 70% IPA.
  c. 2 L plastic beaker.
  d. Male and female Luer lock connectors.
  e. Three-stop PharmaMed tubing.
  f. Extension PharmaMed tubing.
  g. A Blade.
  h. Peristaltic pump.
  i. Autoclaved high vacuum grease.
  j. Big incubator water bath metallic tray.
147. Fill the beaker with 70% IPA and submerge completely the Simple-Flow plates for 5-10 s to sterilize them (Figure 9A).

***Note:*** IPA is required as ethanol 70% brittles the Biomed Clear resin.

148. Remove the excess of 70% IPA with clean paper towels and allow the Simple-Flow device air dry in the cell culture cabinet (Figure 9B).
149. Meanwhile, pour the 70% IPA back into its bottle and prepare the tubing (Figure 9C).
  a. Cut about 20 cm of Extension tubing using a clean blade.
  b. Connect the Extension tubing to the three-stop tubing using male and female Luer lock connectors.
  c. Add female Luer lock connectors at the ends of the tubing.
150. Once the Simple-Flow is dry, screw the female Luer lock connectors into the 3D printed plate (Figure 9D).

***Note:*** Ensure to correctly orient and lock the female Luer connectors into the plate at a 45-degree angle before screwing them securely. Ensure consistency by screwing all connectors in a similar manner.

151. Bring the metallic tray and the peristaltic pump into the cell culture hood. Position the pump on the tray and connect it to the power source.
152. Attach the Three-stop tubing to the cassettes, utilizing two of the three stops.
153. Affix the cassettes to the peristaltic pump with the tubing inserted.
154. Adjust the pressure lever on the cassette to ensure proper flow of liquid through the tubing, usually 2–3 points.
155. Place a standard sterile 24-well cell culture lid onto the Simple-Flow to enclose it. The system is now prepared to start pumping liquid through (Figure 9E).
156. To make the tubing sterile, pump 70% IPA through the system for about 5 min. Use a pump speed of 05.0 to 10.0, i.e. about 320–650 μL/min.
157. Check for bubble formation on the holes inside the Simple-Flow, where the female Luer connector is attached, to make sure that liquid flows through the system (Figure 9F).
158. Once the bubbles disappear, indicating that the tubing is filled with liquid, discard the 70% IPA either by vacuum extraction or pipetting. Decant the plate carefully to better remove the liquid. Repeat this process until no further liquid emerges of the tubing.
159. Repeat steps 156-158 but using DPBS instead to remove the residual IPA.
160. Add 8 mL of BEGM:EGM-2MV medium (2:1 ratio) co-culture medium with 50 nM EC-23 per channel. To be certain that the co-culture medium flows through the tubing, re-examine for bubbles (Figure 9F) and ensure the Luer connectors turn pink (Figure 9G).
161. After confirming the absence of bubbles, adjust pump speed at 00.1 (about 4 μL/min), and use sterile tweezers to insert cell culture inserts containing the co-cultures, initiating hPBECs differentiation under flow conditions.
162. Bring the water bath tray containing the entire setup and place it inside the incubator.^12^

***Note:*** The Peristaltic Pump used in this protocol is IP65-certified meaning that it can withstand incubator conditions. Normal peristaltic pumps should not be placed into incubators.

163. After a few h, once the 3D-printed plate has warmed up within the incubator, inspect for any leaks by observing the presence of pink medium stains on the paper towels.
164. If leaks are found, transfer the entire setup into a cell culture cabinet (troubleshooting). Then:
  a. Carefully further tighten the female Luer lock connectors into the plate.
    ***Note:*** The elevated temperature and moisture levels inside the incubator likely expand the resin material, thus facilitating further tightening of the female Luer lock connectors.
  b. Using sterile cell culture pipette tips, apply autoclaved high vacuum grease along the groove between the plate and the female Luer lock connector.^12^ Ensure thorough coverage of the lower part of the groove to prevent leakage. For optimal application, detach the cassettes from the peristaltic pump and lift the plate for improved visibility.
  c. Replace the kitchen paper towels for new and clean ones.
165. Bring the setup back to the incubator, set pump speed at 00.1, and monitor it for 14 days.

#### Maintenance of co-cultures in the simple-flow

This part of the protocol describes how to change the medium twice a week while culturing primary cells in the Simple-Flow, and how to measure the TEER.

166. Bring the tray containing the entire setup into a sterile cell culture cabinet.
167. Gently decant the Simple-Flow and discard the medium from each channel. Use the reservoirs to remove the medium.
168. Add BEGM:EGM-2MV (2:1 ratio) co-culture medium with 50 nM EC-23 to each channel:
  a. Static 3D-printed plate (no holes): 6 mL/channel.
  b. Flow 3D-printed plate: 8 mL/channel.
169. Bring the entire setup back to the incubator. Set pump speed at 00.1.

***Note:*** Ensure to at least measure TEER on Days 0 (all static conditions), 7 and 14 of ALI.

170. Sterilize and place the TEER machine (EVOM2 Epithelial Voltohmmeter with STX2 electrode) in a cell culture cabinet.
171. Bring the tray containing the entire setup into the cell culture cabinet.
172. Add 150 μL and 500 μL of DPBS + CaCl_2_ + MgCl_2_ in the apical chamber of 6.5 mm and 12 mm inserts respectively.
173. Measure TEER inside the 3D-printed plates.
174. Discard DPBS from inserts, and bring the entire setup back to the incubator.

## Expected outcomes

**Figure 10:**
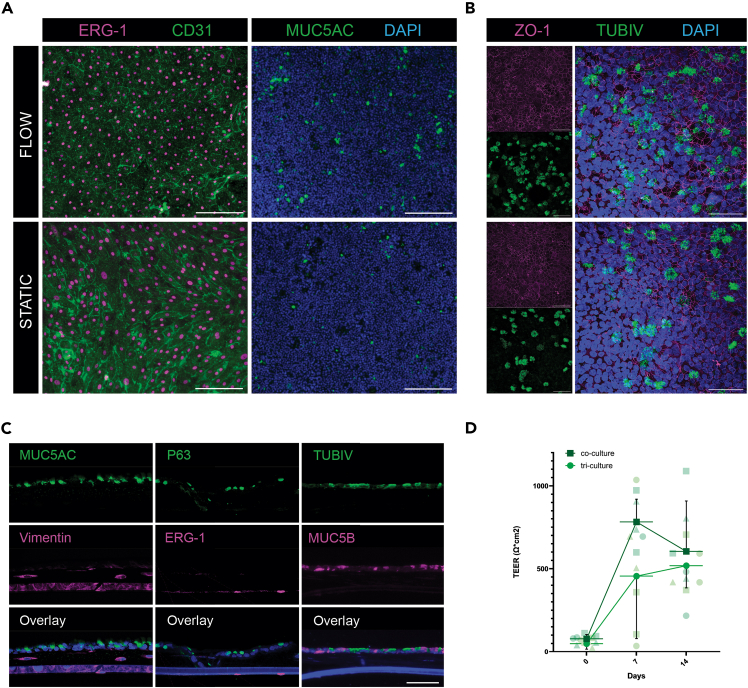
Expected outcomes of the co- and tri-cultures Images of immunofluorescence (IF) staining of hMVECs-hPBECs co-cultures under static or flow conditions. (A) HMVECs expressed endothelial cell markers CD31 (BioLegend 303101, 1:100) and ERG1 (Abcam ab92513, 1:500) after 14 days of being exposed to flow media. HPBECs differentiated to goblet (MUC5AC; Abcam ab3649, 1:500) cells after 14 days at ALI. Scale bar = 200 μm. (B) Images of whole mount IF staining of differentiated HPBECs with ciliated (TUBIV; BioGenex MU178-UC, 1:200) cells, and forming a tight monolayer (ZO-1; Zymed 40-2300, 1:100) after 14 days at ALI, scale bar = 50 μm. (C) Images of IF staining of section of tri-cultures with basal cells (P63), and differentiated ciliated (TUBIV; BioGenex MU178-UC, 1:200) and secretory (MUC5AC; Abcam ab3649, 1:500, and MUC5B; Sigma-Aldrich HPA008246, 1:500) cells, hLFs (Vimentin; Abcam ab92547, 1:500) and hMVECs (ERG1; Abcam ab92513, 1:500)) after 14 days in tri-culture in static conditions. Scale bar = 50 μm. (D) TEER values of hPBECs-HMVECs in co-cultures, and tri-cultures under static conditions.

Here, we described reproducible and reliable protocols for establishing human primary co- and tri-cultures. These cultures can be exposed to flow medium through a 3D-printed flow device called Simple-Flow, for which manufacturing, post-processing and setup protocols were also detailed and reproducible. Upon completion of these protocols, hPBECs co-cultured with hMVECs exhibit differentiation into mucociliary epithelium, while hMVEC maintain their endothelial phenotype for 14 days under both flow and static conditions at ALI. In addition, hPBECs on tri-cultures also show differentiation to ciliated and goblet cells, while hMVECs and hLFs maintain their respective phenotypes for 14 days in static conditions at ALI. Finally, co-cultures and tri-cultures show cell layer integrity (Figure 10).^12^ These co-cultures also have the potential applications in drug testing, and pollution assays, as well as in the study of patient-specific respiratory diseases.

## Limitations

As these protocols rely on cells isolated from tumor-free human lung resection material, access to such material is limited, and donor-to-donor variations are observed in the number of cells isolated, their purity, cell morphology and size, hPBECs mucociliary differentiation, and cell viability. To conduct fluidic experiments, availability to 3D-printers and biocompatible resins is required.

## Troubleshooting

### Problem 1

Endothelial cells detach from PET cell culture insert membranes.

### Potential solution

Ensure to coat inserts ON and include the air-drying step. These two procedures significantly enhanced endothelial cell attachment to PET cell culture insert membranes.

### Problem 2

The type I collagen gels containing the hLFs in the tri-cultures shrink within less than 14 days at ALI.

### Potential solution 1

On day 0 of ALI, or in the event of rapid epansion of hLFs, add 30 nM of the broad-spectrum matrix metalloprotease (MMP) inhibitor GM6001 (a.k.a. Ilomastat, supplier: Tocris, 2983) into the co-culture medium. This inhibits hLFs from further remodeling and degrading collagen matrices.^22^^,^^23^

### Potential solution 2

Immediately before seeding hPBECs, flatten the collagen gels using a sterile medical gauze roll (Figure 7G, supplier: any drug store).

• Open the sterile gauze package.
• Cut the gauze in half with sterile scissors.
• Roll up the gauze with your fingers (Figure 7G).
• Use scissors to cute one side of the gauze roll to ensure uniformity across its surface.
• Gently position the even surface or the rolled-up gauze atop of the collagen gel. Avoid applying excessive pressure to the collagen gel. The primary aim is for the gauze to absorb some of the medium from the gel, thereby flattening it.
• Keep the gauze in an insert for about 5-10 s and gently remove the gauze (Figure 7H).

### Problem 3

Simple-Flow plates exhibit leakages at the junction between the plate and female Luer connectors even when autoclaved grease is applied.

### Potential solution

Identify the source of the leakage, inspect for any holes in the grease between the plate and Luer connectors, and gently adjust the grease to prevent further leakage. If necessary, apply additional grease. In addition, elevate the Simple-Flow by placing it atop a standard cell culture lid or plate to further prevent such leakages. Place paper towels beneath the system to monitor for leaks and maintain cleanliness. When the paper towels come into direct contact with the Simple-Flow, they may absorb any leaked medium. Therefore, it is advisable to place a lid between Simple-Flow and the paper towels to slow down any leakage. Replace paper towels every time they get spotted to continue monitoring for new potential leaks.

### Problem 4

High bacterial infection rates of isolated lung cells.

### Potential solution

Bacterial infections are frequently encountered during cell isolation processes, especially because the lungs are extensively exposed to pathogens and pollutants. Throughout the isolation, purification, and expansion stages – prior to the initial cell freezing – ensure to use antibiotics P/S and GA-1000. Post-freezing, using P/S antibiotics alone should suffice. In addition, ensure optimal sterility conditions for all cell culturing conditions and equipment, especially on the day of cells isolation.

## Resource availability

### Lead contact

Further information and requests for resources and reagents should be directed to and will be fulfilled by the lead contact, Robbert J. Rottier (r.rottier@erasmusmc.nl).

### Technical contact

Technical questions on executing this protocol should be directed to and will be answered by the technical contact, Cinta Iriondo (m.iriondomartinez@erasmusmc.nl).

### Materials availability

This study did not generate new materials.

### Data and code availability

No additional data were used, and this paper does not report original code.

## Acknowledgments

This work was supported by grants from the Human Disease Modeling Award of the Erasmus MC (FB380799; C.I.), the Erasmus MC-Erasmus University-TU-Delft Flagship (“Decoding Real-Time, Personalized Health Impact of Climate Change and Pollution”; S.K.), and ZonMw (114025011; K.-P.S. and R.J.R.). The graphical abstract and illustrations were created using Biorender.com.

## Author contributions

Conceptualization, R.J.R., C.I., S.K., and K.-P.S.; methodology, R.J.R., C.I., S.K., and K.-P.S.; validation, C.I.; formal analysis, R.J.R. and C.I.; investigation, R.J.R., C.I., S.K., K.-P.S., M.B.-v.K., and A.B.-d.M.; resources, R.J.R.; writing – original draft, C.I.; writing – review and editing, R.J.R., C.I., S.K., K.-P.S., M.B.-v.K., and A.B.-d.M.; visualization, R.J.R. and C.I.; supervision, R.J.R.; project administration, R.J.R. and C.I.; funding acquisition, R.J.R.

## Declaration of interests

The authors declare no competing interests.
